# Response to S. Leonard Syme’s Essay

**Published:** 2004-03-15

**Authors:** Robert G. Robinson

**Affiliations:** Office on Smoking and Health, Centers for Disease Control and Prevention

To the Editor:

The recent essay by Dr Leonard Syme contributes constructively to the dialogue on disparities (1). It validates the idea that the traditional focus on the individual and risk factors is limited and underscores the importance of environment and community. The complexity of community, however, is not apparent in the essay, and this oversight adds to deficiencies in interventions. Public health needs a different paradigm for assessment and intervention development.

One barrier mentioned by Dr Syme is arrogance, and to that I would add elitism; both prevent experts from relating and adopting paradigms that use community as the unit of analysis. Both challenge diversity and inclusivity, which are necessary for community partnerships. Also troublesome is a limited definition of competency. Dr Syme illustrates the ineffectiveness of interventions in several studies. Others have outlined limitations in addressing community: McKenzie (2) (on the impact of racism and community), Vena and Weiner (3) (on the social determinants of health and community), and Richards, Kennedy, and Krulewitch (4) (on evaluation models that insufficiently encompass community complexity).

Dr Syme uses environment as a metaphor for community, but environmental change is safe verbiage that disguises the limitations of theory and practice. Environmental change factors are merely risk factors writ large. They are reductionist, failing to build a comprehensive understanding of community and reinforcing traditional analyses, which assess outcomes in terms of etiology or predictive factors. They do not assess relationship to community but impose it. Because risk factors relate to individual well-being, we often incorrectly assume they relate to community outcomes.

Dr Syme also uses social status as a metaphor for community. The construct is simple: draw a circle around an entity and name it community. Indeed, Dr Syme defines as community any group that is targeted: citizens of Richmond, Calif, fifth-graders, bus drivers. Each possesses an ethos and a consciousness, but each also lacks the complexity of community. The most critical mistake in targeting a social stratum is creating the illusion that we are targeting a community. We design an intervention for welfare mothers, for example, and write up our findings as a community intervention. But targeting the poor is not the same thing as targeting the community. Change theory derives from the individual unit of analysis and from constructs that do not reflect the complexity inherent in communities.

Another flaw in Dr Syme's essay is the exclusion of race/ethnicity. This exclusion is compounded by insufficiency of community theory and practice and emphasis on etiology and risk factors. Multivariate analysis suggests variables that are important based on statistical significance. Education and income knock race/ethnicity "out of the box." This exclusion is incorrect. Etiology assumes a core role in developing interventions. This may make sense when the unit of analysis is the individual, but it is unfounded when the target is the community.

Communities defined by race/ethnicity magnify the error. Although poverty is the predictive variable, poor people tend not to live in integrated communities. The social reality of imposed segregation is ignored. Indeed, observations of an area of homelessness in Los Angeles showed that white, black, and Latinos each reside on separate street corners (5).

We must develop interventions at 2 levels: by identifying causal factors and deciding at what depth the intervention is to occur and by relating the causal factors to the target population. What do causal factors mean to the population? What is the best protocol for delivery? Superimposing the community over the multivariate analysis is a paradigm shift from traditional biostatistical training, and we need to explore it.

The challenge for the 21st century is to develop theory and practice that resonate with community and its determinants: history, culture, context, and geography. Community competence, a protocol for intervention development, is one solution (6). It avoids the reductionism inherent in cultural competency, and is enhanced by language, literacy, positive imagery, salient imagery, multiple generations, and diversity.

Progress in public health science and practice throughout the 20th century reflects our understanding of the individual. While progress in environmental health has been obvious, progress within race/ethnic communities is not so evident. Upgrading our sanitation and related regulatory protocols benefited populations defined by geography and work site. African Americans and Native Americans continue to demonstrate disparities. Ethnic communities within Latino and Asian/Pacific Islander aggregations demonstrate similar disparities. Why? Our science and practice fails to assess community trends or develop tailored interventions. The 21st century should be the "century of the community," and the emphasis of efforts to improve theory and practice ought to reflect this paradigm.
